# Singular Case of an Omental Hernia

**Published:** 1799-04

**Authors:** James Stratton

**Affiliations:** Swedesburgh, New Jersey


### *Singular Cafe of an Omental Hernia:—By Dr.* JAMES STRATTON, *of Swedefburgh, New Jerfey.*

ON the 26th of April, 1795, W. C. of the county of Salem, in New Jerfey, aged 45, of a firm conftitution, was attacked with a violent colic, On the 28th his bowels were freely opened, and fome relief was obtained by injection: 30th, the bowels continued open, but very uneafy; a hiccup, or a kind of belching, was obferved, which frequently brought up a part of the contents of the ftomach. On examination, a tumour was found in the right groin, of about the fize of two fingers, owing to the protrufion of a portion of the omentum through the ring in the abdominal mufcles, forming an omental hernia.

After

After plentiful bleeding, attempts were made to reduce the protruded part, without effect; the tumor being hard, and very *tender* *, cold saturnine applications were then industriously employed, and continued for several days; during which time, several attempts were made to reduce it, but with no better success. The patient having refused, after repeated solicitations, to submit to any operation, remained in nearly the same state until about the 10th of May, being two weeks after the attack, when the tumour subsided, and became soft, insensible, and crepitous; at the same time some appearance of tumour was observed in the most depending part of the scrotum, accompanied with pain. This increased rapidly until the 14th, when the tumour, having attained the size of a man's head, burst, pouring out a quantity of pus mixed with fetid sanies. The strangulated and sphacelated parts of the omentum having been separated from the sound, after destroying the hernial sac, had fallen down into the scrotum, producing inflammation, and suppuration sufficient to effect their discharge. The abscess in a few days began to heal, and, by simple dressings only, the patient recovered, and is now living, without experiencing any inconveniency from what has happened.

* We suppose the author means, *sensible to the touch.*

